# Diabetes and nephrotic syndrome: Questions

**DOI:** 10.1007/s00467-016-3558-3

**Published:** 2017-01-10

**Authors:** Rodney D Gilbert, Edward Hind, Bhumita Vadgama

**Affiliations:** 1 Southampton Children’s Hospital–University of Southampton School of Medicine, Tremona Road, Southampton, SO16 6YD UK; 2 Hampshire Hospitals NHS Foundation Trust, Basingstoke, UK; 3 Department of Cellular Pathology, University Hospital Southampton NHS Foundation Trust, Southampton, UK

**Keywords:** Diabetes, Nephrotic syndrome, Poor stamina, Abdominal pain, Short stature, Focal segmental glomerulosclerosis

## Case summary

A 17-year-old girl was referred for evaluation of proteinuria. A diagnosis of diabetes had been made 2 years earlier after a short history of polyuria, polydipsia, weight loss and tiredness. A presumptive diagnosis of type 1 diabetes was made based on the short history, blood glucose concentration of 14.9 mmol/L and significant ketosis at presentation. Diabetic control had been excellent, and in fact the insulin dose had required reduction.

Six months after the diagnosis of diabetes her urine was tested for protein as part of routine diabetes monitoring. Glycated haemoglobin (HbA_1_C) levels were low at 4.8% (29 mmol/mol). Early morning urine protein/creatinine ratios were 282, 268 and 170 mg/mmol. Plasma electrolytes, urea, creatinine, calcium, phosphate and parathyroid hormone levels were all normal, as were plasma complement C3 and C4 concentrations.

The past medical history was notable for poor stamina which had forced her to give up playing soccer several years previously. Since the onset of diabetes she had begun to suffer from pain in the lower abdomen and knees. The symptoms were very distressing and interfered with sleep and socialising. She was seen by multiple specialists and had undergone numerous tests, including knee radiographs, abdominal magnetic resonance scans and numerous blood tests. No diagnosis was made.

The patient was the younger of two children born to healthy, unrelated parents. There was no family history of diabetes, deafness or renal disease. Her height was 145.3 cm (*Z* score −3.01) and her weight was 42.4 kg (*Z* score −2.25). There was no oedema. Her systolic/diastolic blood pressure was 98/68 mmHg. Her abdomen was not distended and was soft to palpation. There was generalised tenderness but no masses or peritonism. The rest of the examination was unremarkable.

**Fig. 1:**
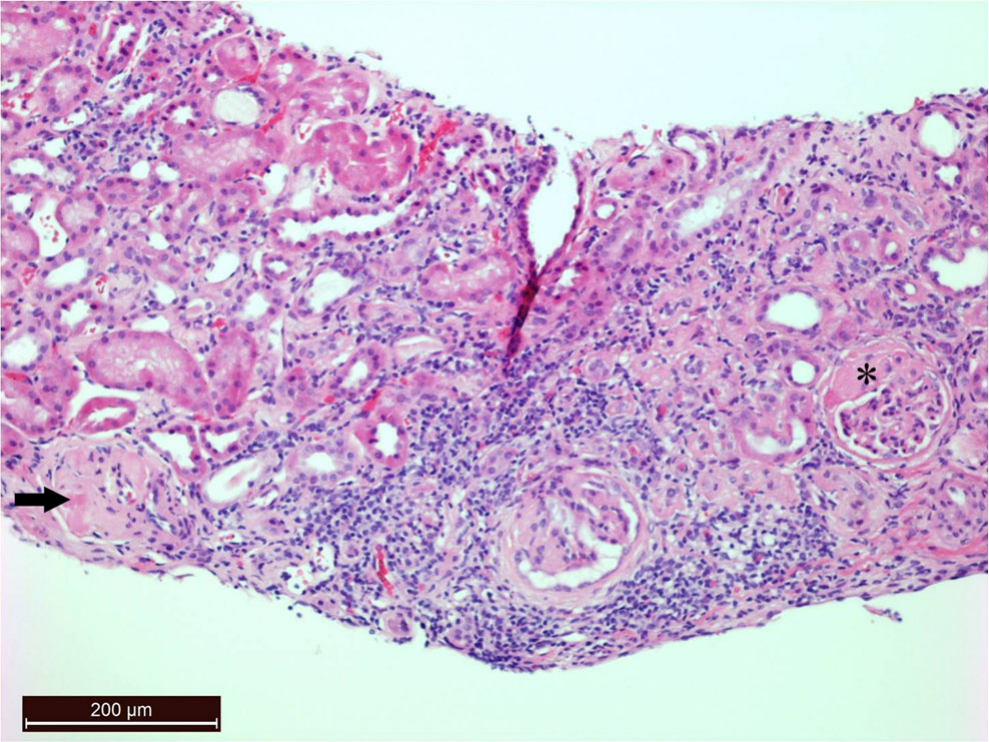
Haematoxylin and eosin stain (magnification ×100) photomicrograph showing globally sclerosed (*arrow*) and segmentally sclerosed (*asterisk*) glomeruli. The central glomerulus demonstrates periglomerular fibrosis. There is also mild interstitial chronic inflammation and some tubular atrophy.

Laboratory tests showed a normal full blood count and a slightly raised erythrocyte sedimentation rate of 28 mm/h, but a normal C-reactive protein concentration of <1 mg/L. The plasma creatinine was 59 μmol/L, giving an estimated glomerular filtration rate of 86 ml/min/1.73 m^2^. The urine protein/creatinine ratio was markedly elevated at 483 mg/mmol (normal <23 mg/mmol; nephrotic range >200 mg/mmol), and the plasma albumin concentration was slightly reduced at 31 g/L. The HbA_1_C level was low at 34 mmol/mol and the C-peptide level was 1046 pmol/L. Tests for islet cell and glutamic acid decarboxylase antibodies were negative. Audiometry showed that the patient had normal hearing. The renal biopsy showed 40 glomeruli, of which 13 were globally sclerosed and five had segmental sclerosis (Fig. 1). There was no nodular glomerulosclerosis and immunostaining was negative.

## Questions

1. How are the diabetes and renal disease connected to each other, if at all?
2. What further investigations should be undertaken?
3. What is the diagnosis?

